# Impact of SARS-CoV-2 infection on clinical outcomes of *in vitro* fertilization treatments: a systematic review and meta-analysis

**DOI:** 10.3389/fendo.2023.1233986

**Published:** 2023-10-06

**Authors:** Yamei Xue, Yuping Xiong, Xiaohong Cheng, Kun Li

**Affiliations:** ^1^ Assisted Reproduction Unit, Department of Obstetrics and Gynecology, Sir Run Run Shaw Hospital, School of Medicine, Zhejiang University, Hangzhou, Zhejiang, China; ^2^ School of Pharmacy, Hangzhou Medical College, Hangzhou, Zhejiang, China; ^3^ School of Basic Medical Sciences and Forensic Medicine, Hangzhou Medical College, Hangzhou, Zhejiang, China; ^4^ Zhejiang Provincial Laboratory of Experimental Animal’s & Nonclinical Laboratory Studies, Hangzhou Medical College, Hangzhou, Zhejiang, China

**Keywords:** coronavirus disease 2019 (COVID-19), severe acute respiratory syndrome coronavirus 2 (SARS-CoV-2), *In vitro* fertilization (IVF), clinical outcome, meta-analysis, infertility, male, female

## Abstract

The influence of SARS-CoV-2 infection on clinical outcomes in patients undergoing *in vitro* fertilization has been uncertain. Therefore, this systematic review and meta-analysis aimed to evaluate the impact of past SARS-CoV-2 infection on IVF outcomes. A comprehensive search of PubMed, EMBASE, and Cochrane Library databases was conducted from December 2019 to January 2023. Included studies comparing IVF outcomes between patients with prior SARS-CoV-2 infection and controls without previous infection were analyzed. Study quality was assessed using the Newcastle-Ottawa Quality Assessment Scale. Sensitivity analysis, publication bias, and heterogeneity were also examined. The review protocol was registered with PROSPERO (CRD42023392007). A total of eight studies, involving 317 patients with past SARS-CoV-2 infection and 904 controls, met the inclusion criteria. The meta-analysis revealed no significant differences between the infection group and controls in terms of clinical pregnancy rate (OR 0.97, 95% CI 0.73-1.29; *P* = 0.82), implantation rate (OR 0.99, 95% CI 0.67-1.46; *P* = 0.96), or miscarriage rate (OR 0.64, 95% CI 0.15-2.65; *P* = 0.53). Subgroup analyses based on transfer type demonstrated comparable clinical pregnancy rates between the two groups in both fresh embryo transfer (OR 0.97, 95% CI 0.69-1.36; *P* = 0.86) and frozen embryo transfer (OR 0.96, 95% CI 0.38-2.44; *P* = 0.94). In conclusion, this meta-analysis suggests that previous SARS-CoV-2 infection does not have a detrimental impact on clinical outcomes in IVF patients. These findings provide valuable insights into assessing the influence of prior SARS-CoV-2 infection on successful pregnancy outcomes in IVF treatment. The systematic review was performed based on the Preferred Reporting Items for Systematic Reviews and Meta-Analyses (PRISMA) statement. This review was prospectively registered with the International Prospective Register of Systematic Reviews (ID CRD42023392007) on January 16, 2023.

## Introduction

Coronavirus disease 19 (COVID-19) caused by severe acute respiratory syndrome coronavirus-2 (SARS-CoV-2) infection, has led to a serious and expanding pandemic around the world. The entry of coronavirus into host cells depends on angiotensin-converting enzyme 2 (ACE2), a cellular receptor, and transmembrane protease serine-2 (TMPRSS2), a cellular protease (1–3). This has raised concerns about the potential impact of SARS-CoV-2 infection on organs with high ACE2 or TMPRSS2 expression that may be more vulnerable to adverse sequelae due to infection (4).

SARS-CoV-2 infection has been implicated in various aspects of human fertility. In the male, ACE2 or TMPRSS2 expressed in spermatogonia, peritubular myoid cells, and testicular somatic cells in the testis tissue (5–7); and in some studies, semen parameters were significantly decreased in mildly and moderately infected patients after coronavirus infection, compared to before infection (8, 9). In females, ACE2 and TMPRSS2 are co-expressed in the ovarian cortex, medulla, oocytes (10, 11), endometrium (12, 13), the membrane of trophectoderm, hypoblast, and epiblast cells in blastocysts (14); ACE2 and TMPRSS2 co-expression increased with oocyte maturity (15); and ACE2 is expressed in all stages of follicular maturation in the human ovary (16). Furthermore, SARS-CoV-2 infection has been associated with ovarian dysfunction, disturbs the follicular microenvironment, potentially affects reproductive outcomes in the study (17), and potentially interferes with embryo implantation and pregnancy. Besides, medium, or high SARS-CoV-2 IgG levels in follicular fluid are associated with a lower number of retrieved oocytes (17).

Therefore, it is necessary to evaluate the potential risks of SARS-CoV-2 infection *in vitro* fertilization (IVF). Although the effect of SARS-CoV-2 infection on clinical outcomes of *in vitro* fertilization has been reported, a small sample size was employed in most studies. Meanwhile, the evidence on the effect of SARS-CoV-2 infection on the clinical outcomes and fertility of patients undergoing IVF treatment has not been systematically reviewed. In this study, we aimed to perform a systematic review and meta-analysis to present a comprehensive summary of the available evidence of the effect of SARS-CoV-2 infection on the clinical outcomes of patients undergoing IVF treatment. This study provides valuable insights to evaluate the potential impact of SARS-CoV-2 infection on reproductive outcomes in patients undergoing IVF treatment.

## Methods

The systematic review was performed based on the Preferred Reporting Items for Systematic Reviews and Meta-Analyses (PRISMA) statement. This review was prospectively registered with the International Prospective Register of Systematic Reviews (ID CRD42023392007) on January 16, 2023.

### Search strategy

PubMed, EMBASE and Cochrane Library were searched from December 1, 2019, to January 15, 2023, using a search strategy that combined Medical Subject Heading (MeSH) and EMTREE terms. The target terms included ‘‘fertilization *in vitro*’’, “IVF”, “*in vitro* fertilization”, “intracytoplasmic sperm injection”, “ICSI”, “coronavirus disease 2019”, “COVID-19”, “severe acute respiratory syndrome coronavirus 2”, “SARS-CoV-2”, “infection”, “outcome”, “pregnancy”. The search terms were combined using Boolean operators AND, OR, and NOT. We applied filters to exclude irrelevant articles and ensure the search’s reproducibility.

### Eligibility criteria

1. population: this review focused on patients who had a history of SARS-CoV-2 infection and underwent IVF treatment. Studies included in this review were required to report clinical outcomes after embryo transfer for both the infection population and non-infection population.
2. Exposure: patients underwent routine serum SARS-CoV-2 antibody tests and/or reverse transcription-polymerase chain reaction (RT-PCR) tests for detecting SARS-CoV-2 RNA at least one time. The COVID group included patients with a positive test before IVF treatment and the control group referred to those patients who have no history of COVID infection.
3. Outcomes: the primary outcome was the clinical pregnancy rate. The secondary outcomes included early miscarriage rate and implantation rate. Studies that reported any of the outcomes above were included in this review.
4. Setting and language: this review did not restrict settings and languages.
5. Study design: all observation studies (case-control studies, cohort studies, and cross-sectional studies) will be included.
6. Exclusion: this review excluded case reports, case series, reviews without original data presented, commentaries, and editor letters. Studies involving preimplantation genetic testing (PGT), oocyte or sperm donation cycles were excluded from this review. Studies that only provided the outcome percentages, rather than the absolute values of each group were excluded as well.

### Study selection

Two reviewers (YMX and YPX) independently assessed the titles and abstracts of all records. Full-text studies of selected citations were used to assess the eligibility. Each study was included or excluded according to the inclusion and exclusion criteria. Any discrepancies were resolved through discussion with a third reviewer (KL).

### Data extraction

Data were extracted independently by two reviewers (YMX and YPX), and controversial data were discussed and agreed on. The information collected included publication date, authors, study period, location, study design, setting, sample size, time of COVID-19 diagnosis, methods of COVID-19 detection, the severity of COVID-19, transfer type, and clinical outcomes. When data were analyzed by subgroups (e.g., fresh and frozen embryo transfer) in the studies, the extracted data were pooled for the overall meta-analysis.

### Outcome measures

The primary outcome was the clinical pregnancy rate, which was defined as the observation of a gestational sac with fetal heartbeat on ultrasound imaging divided by the number of transfers. The secondary outcomes included early miscarriage rate and implantation rate. The early miscarriage rate was defined as the loss of pregnancy within the first three months divided by the number of clinical pregnancies. The implantation rate was defined as the number of gestational sacs observed divided by the number of embryos transferred.

### Quality assessment

The Newcastle-Ottawa Scale (NOS) was used to assess the methodological quality of included studies (18, 19). Two reviewers (YMX and YPX) independently accessed the quality of included comparative cohort studies. The major three domains (eight items) of bias to be assessed consist of selection (items: representativeness of the exposed cohort, selection of non-exposed cohort, and ascertainment of the exposure), comparability (item: comparability based on the study design or analysis), and ascertainment of outcome (items: assessment of the outcome and statistical test). A maximum of one star could be assigned for every item under the selection, ascertainment of outcome, and exposure domain. A maximum of two stars could be assigned for the items under the section of comparability. NOS quality assessment scored more than or equal to 7 as high quality, 4-6 as medium quality, and <4 as low quality. Any unresolved disagreements were evaluated by a third reviewer (KL).

### Statistical analysis

Meta-analyses were performed by RevMan ver. 5.4.1 software (Cochrane Collaboration, Copenhagen, Denmark). The Mantel-Haenszel method was used for dichotomous variable data (clinical pregnancy rate, implantation rate, and early miscarriage rate), which were presented as odds ratios (OR) with a two-sided 95% confidence interval (CI). Statistical heterogeneity was assessed by the value of I^2^ index and Q test. I^2^ values <50% and *P* value of χ2 test >0.10 were considered to have low heterogeneity and fixed effects models were used. When I^2^ values >50% and a *P* value of χ^2^ test < 0.10 were considered to indicate moderate to high heterogeneity, a random effects model was used to analyze the data (20, 21). *P* < 0.05 was considered statistically significant. Subgroup analyses were performed according to the transfer type: fresh embryo transfer and frozen embryo transfer.

Furthermore, to evaluate the robustness of the effect size, we conducted sensitivity analyses by excluding each study (Leave-one-out meta-analysis) to explore the impact of individual studies on the pooled effect size. Potential publication bias was examined using the symmetry of the funnel plot and the Egger regression test (22). Statistical analyses were performed with Stata version 17.0 (StataCorp LLC, College Station, TX, USA).

## Results

### Literature screening process

**Figure 1:**
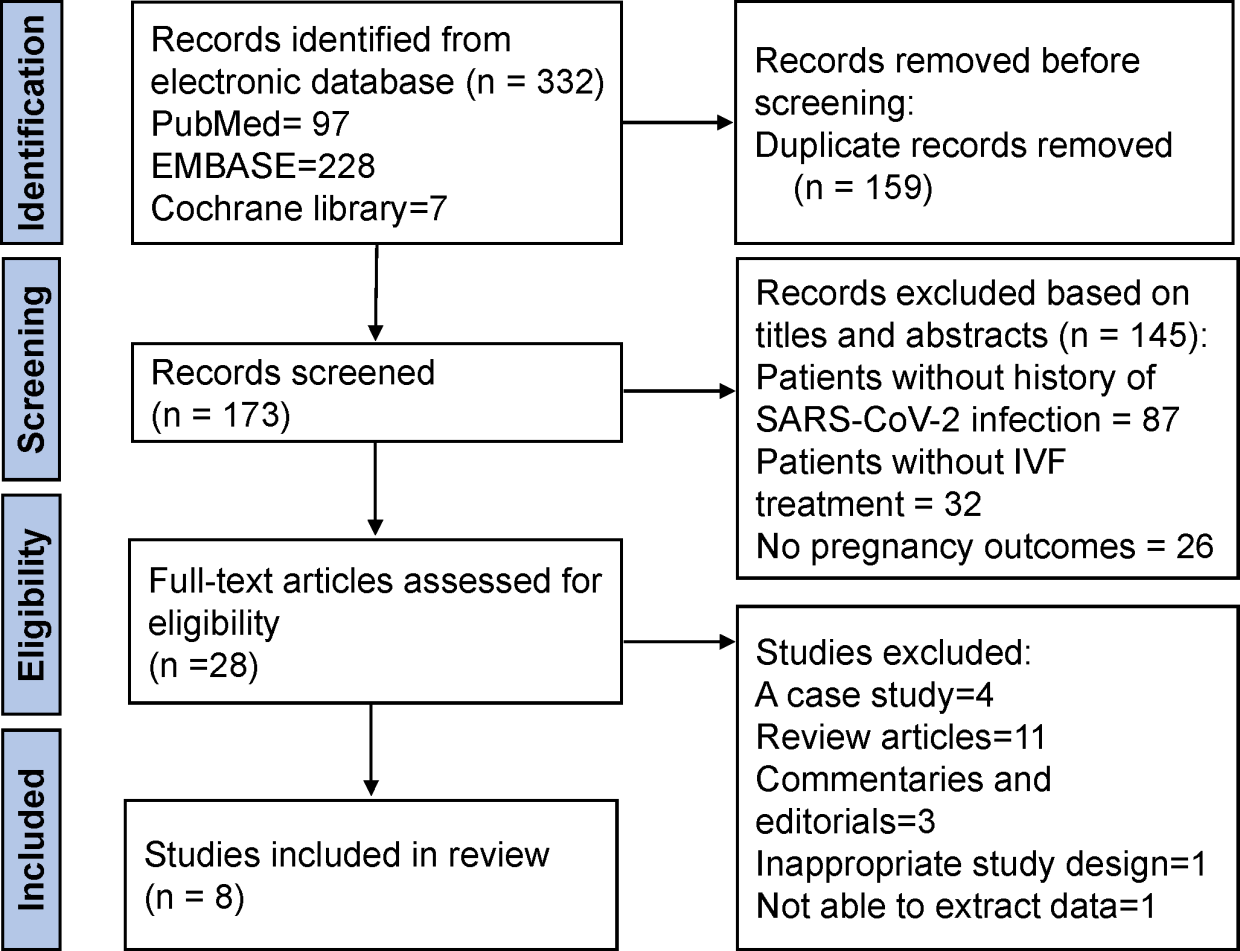
Flowchart of the study selection process.

A flowchart of the literature screening process is shown in **Figure 1**. Our search identified 97 PubMed, 228 EMBASE, and 7 Cochrane Library records. A total of 332 reports were searched; 159 were duplicates, leaving 173 reports. Based on eligibility criteria, after being screened for titles and abstracts, 145 articles were excluded for the following reasons: patients without a history of SARS-CoV-2 infection (n = 87), patients without IVF treatment (n = 32), and no clinical pregnancy outcomes (n = 26). Twenty-eight full articles were obtained to assess its eligibility. Twenty of them were excluded: 4 were case studies, 11 were reviews, 3 were commentaries and editorials, one contained inappropriate study design, and one lacked extract data. Therefore, eight studies were finally included in this review.

### Study characteristics

There was a total of 317 patients with past COVID-19 infection and 904 controls, included in 8 studies (23–30). The characteristics of the included studies are presented in **Table 1**. The publication date of included studies varied from 2021-2023. Of them, one was a prospective observational study, and the remaining 7 were retrospective cohort studies. Two of the 8 included studies were multicenter studies, and the rest were single-center studies. Five studies transferred fresh embryos, 2 transferred frozen-thawed embryos, and 1 transferred fresh and frozen embryos. All included studies have reported the primary outcomes. Regarding secondary outcomes, 4 and 3 studies reported implantation rate and early miscarriage rate, respectively.

**Table 1:** Characteristics of included studies.

Author, Y (study period)	City, country	Study design	Setting	Time of diagnosis	Diagnosis COVID-19*	Severity of COVID-19	The number of patients included		Type of transfer	Reference
							Infection	Control		
Wang M et al., 2021(2020.5-2021.1)	Wuhan, China	Retrospective cohort study	Single-center	At least 4 months between the first diagnosis and IVF treatment	RT-PCR and antibody	Asymptomatic or mild	65	195	Fresh embryo transfer	(23)
Albeitawi S et al., 2022(2021.9-2021.11)	Irbid, Jordan	Retrospective study	Multicenter	NA	NA	NA	52	98	Fresh embryo transfer	(24)
Youngster M et al., 2022 a (2021.1-2012.6)	Zerifin and Herzliya, Israel	Retrospective cohort study	Multicenter	The time interval from infection to oocyte retrieval: 8-348 days	NA	Asymptomatic or mild	121	121	Fresh embryo transfer	(25)
Wang M et al., 2022(2020.5-2021.1)	Wuhan, China	Retrospective cohort study	Single-center	Three diagnostic times: the first visit, before the COH procedure, and before oocyte retrieval	RT-PCR and antibody	Asymptomatic, mild, or moderate	50	148	Fresh embryo transfer	(26)
Youngster M et al., 2022 b (2021.1-2021.6)	Zerifin, Israel	Retrospective cohort study	Single-center	Within 1 year before embryo transfer	RT-PCR	NA	41	41	Frozen embryo transfer	(27)
Aizer A et al., 2022(2021.1-2021.8)	Tel Aviv, Israel	Retrospective cohort study	Single-center	NA	NA	NA	26	234	Frozen embryo transfer	(28)
Braga DPAF et al., 2022(2019.3-2021.6)	Sao Paulo, Brazil	Historical cohort study	Single-center	Within 6 months before IVF treatment	Antibody	NA	22	66	Fresh embryo transfer	(29)
Adler Lazarovits C et al., 2023(2021.10-2021.11)	Jerusalem, Israel	Prospective observational study	Single-center	NA	RT-PCR	NA	21	13	Fresh and frozen embryo transfer	(30)

*Three methods for diagnosis of COVID-19: RT-PCR, antibody, and other.

COVID-19, coronavirus disease 19; IVF, in vitro fertilization; RT-PCR, reverse transcription-polymerase chain reaction; NA, not available.

### Quality of included studies

**Figure 2:**
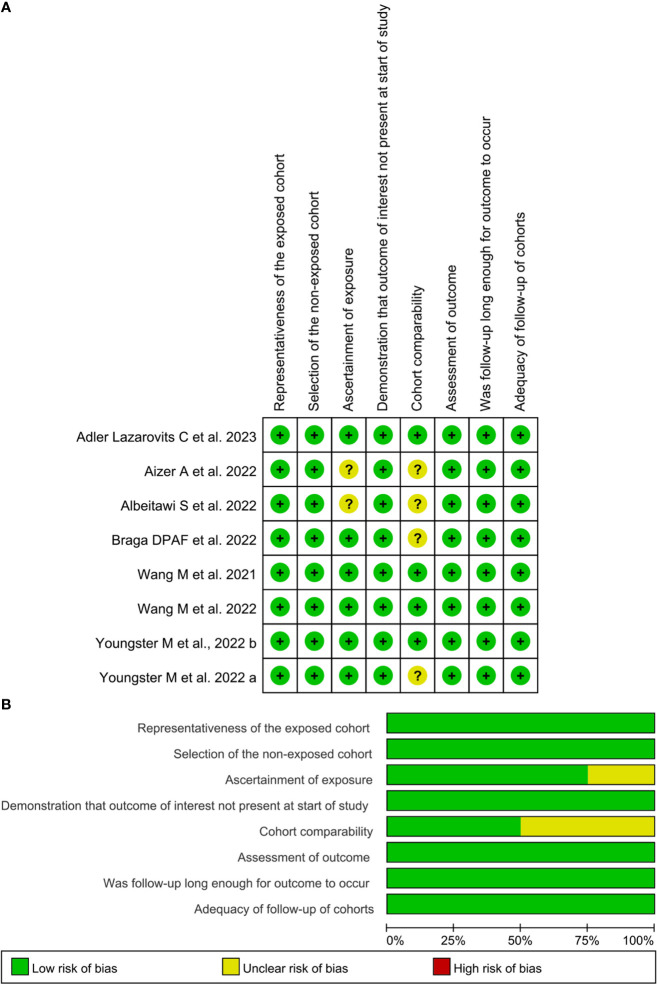
Risk of bias of included studies based on the Newcastle-Ottawa Scale. **(A)**, the Newcastle-Ottawa Scale rating per study; **(B)**, Summative Newcastle-Ottawa Scale rating. Images **(A, B)** were generated with RevMan Version 5.4.

**Table 2** shows the NOS quality scores of the included studies. Overall, 6 of the 8 cohort studies (23, 25–27, 29, 30) were of high quality (NOS score ≥ 7 stars), whereas the remaining two studies (24, 28) scored 6 and were considered medium quality (**Figures 2A, B**). In the selection domain of NOS, 6 of the 8 included studies scored 4 stars (23, 25–27, 29, 30). The study by Albeitawi et al. (24) and the study by Aizer et al. (28) scored 3 stars because the two studies did not provide the time of COVID-19 diagnosis and the methods of COVID-19 detection.

**Table 2:** Quality assessment of included studies based on the Newcastle-Ottawa Scale.

Study (author, y)	Selection				Comparability	Outcome			Quality score
	Representativeness of the exposed cohort	Selection of Non-exposed cohort	Ascertainment of exposure	Demonstration that the outcome of interest was not present at the start of the study	Comparability of cohorts based on the design or analysis ^#^	Assessment of outcome	Was follow-up long enough for outcomes to occur?	Adequacy of the follow-up of cohorts	
Wang M et al., 2021 (23)	a)*	a)*	a)*	a)*	c)**	a)*	a)*	a)*	9
Albeitawi S et al., 2022 (24)	a)*	a)*	d)	a)*	d	a)*	a)*	a)*	6
Youngster M et al., 2022 a (25)	a)*	a)*	b)*	a)*	a)*	a)*	a)*	a)*	8
Wang M et al., 2022 (26)	a)*	a)*	a)*	a)*	c)**	a)*	a)*	a)*	9
Youngster M et al., 2022 b (27)	a)*	a)*	a)*	a)*	c)**	a)*	a)*	a)*	9
Aizer A et al., 2022 (28)	a)*	a)*	d)	a)*	d	a)*	a)*	a)*	6
Braga DPAF et al., 2022 (29)	a)*	a)*	a)*	a)*	a)*	a)*	a)*	a)*	8
Adler Lazarovits C et al., 2023 (30)	a)*	a)*	b)*	a)*	c)**	a)*	a)*	a)*	9

Representativeness of the exposed cohort: a) truly representative of the average in vitro fertilization therapy patients in the community, b) somewhat representative of the average in vitro fertilization therapy patients in the community, c) selected group of users, d) no description of the derivation of the cohort. Selection of non-exposed cohort: a) drawn from the same community as the exposed cohort, b) drawn from a different source, c) no description of the derivation of the non-exposed cohort. Ascertainment of exposure: a) secure record, b) structured interview, c) written self-report, d) no description. Demonstration that outcome of interest was not present at the start of the study: a) yes, b) no. Comparability of cohorts based on the design or analysis: a) study controls for female’s age, b) study controls for any additional factors, c) study controls for female’s age and other factors, d) no study controls for female’s age or any other factors. Assessment of outcome: a) independent blind assessment, b) record linkage, c) self-report, d) no description. Was follow-up long enough for outcomes to occur: a) yes, b) no. Adequacy of follow-up of cohorts: a) complete follow-up, b) subjects lost to follow-up unlikely to introduce bias - small number lost ≥ 90% follow-up, c) follow-up rate <90% and no description of those lost, d) no statement.

^#^ A maximum of 2 stars can be allotted in this category, one for female age, the other for other controlled factors. *, a maximum of one star could be assigned; **, a maximum of two stars could be assigned.

In the comparability item of NOS, 4 studies (23, 26, 27, 30) scored 2 stars, respectively because the studies provided the controls matched with females’ age and other parameters. The study by Youngster et al. (25) and the study by Braga et al. (29) scored one star, respectively because the controls were exclusively matched with females’ age. The study by Albeitawi et al. (24) and the study by Aizer et al. (28) did not obtain a star because the control groups reported in these two studies did not match the age of the females.

### Sensitivity analysis

**Figure 3:**
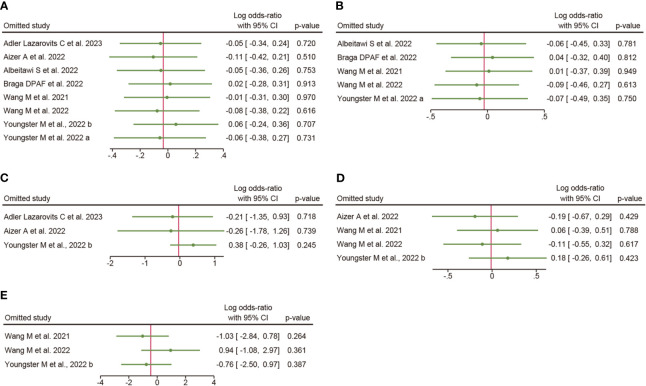
Sensitivity analysis. **(A)**, overall clinical pregnancy rate; **(B)**, clinical pregnancy rate in fresh embryo transfer; **(C)**, clinical pregnancy rate in frozen embryo transfer; **(D)**, implantation rate; **(E)**, early miscarriage rate.

The results of sensitivity analyses are shown in **Figure 3**: the overall clinical pregnancy rate (**Figure 3A**), clinical pregnancy rate in fresh embryo transfer (**Figure 3B**), clinical pregnancy rate in frozen embryo transfer (**Figure 3C**), implantation rate (**Figure 3D**), and early miscarriage rate (**Figure 3E**). The results indicated that excluding any single study had no significant effect on the total effect size (All *P* value > 0.05).

### Publication bias

**Figure 4:**
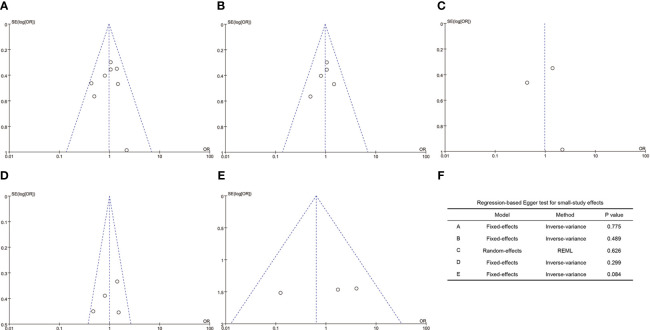
Funnel plots and Egger’s test. **(A)**, overall clinical pregnancy rate; **(B)**, clinical pregnancy rate in fresh embryo transfer; **(C)**, clinical pregnancy rate in frozen embryo transfer; **(D)**, implantation rate; **(E)**, early miscarriage rate; **(F)**, values of the Egger test.

As indicated in **Figure 4**, the funnel plots of the A to E are not asymmetrical and were evenly vertically distributed, demonstrating no or limited publication bias. The results of the Egger test (**Figure 4F**) showed that there was no publication bias in the overall clinical pregnancy rate, clinical pregnancy rate in fresh embryo transfer, clinical pregnancy rate in frozen embryo transfer, implantation rate, and early miscarriage rate, with Egger values of 0.775, 0.489, 0.626, 0.299, and 0.084, respectively.

### Primary outcomes

#### Clinical pregnancy rate

**Figure 5:**
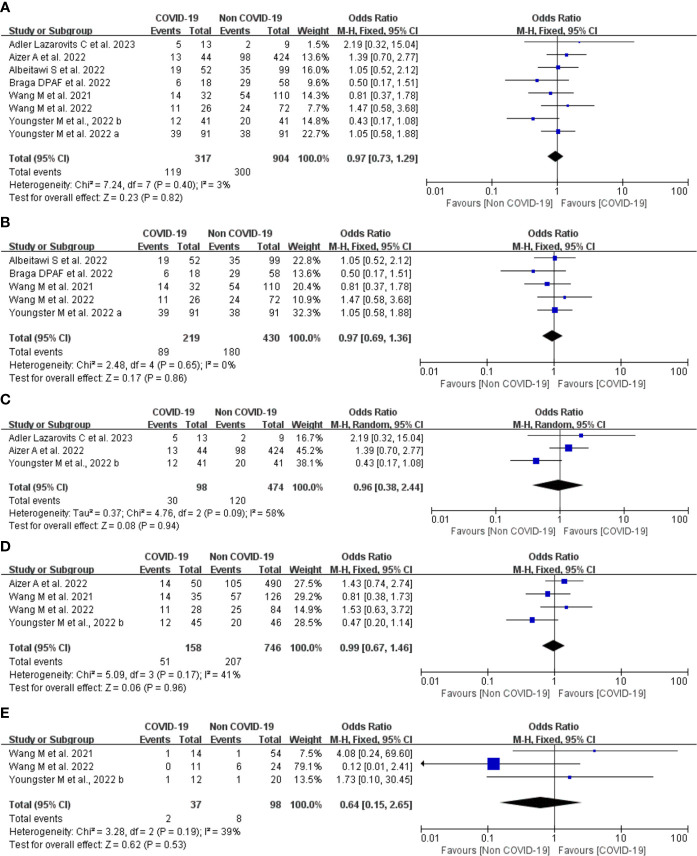
Forest plot of studies of COVID-19 vs. non-COVID-19 for the clinical outcomes. **(A)**, overall clinical pregnancy rate; **(B)**, clinical pregnancy rate in fresh embryo transfer; **(C)**, clinical pregnancy rate in frozen embryo transfer; **(D)**, implantation rate; **(E)**, early miscarriage rate; M-H = Mantel-Haenszel method.

A total of eight studies involving 317 patients with COVID-19 infection and 904 controls undergoing IVF treatment reported the clinical pregnancy rate. Overall, the Q test and I^2^ index showed low heterogeneity between the two groups (*P* = 0.40, I^2 = ^3%), and fixed-effects model analysis was used. The meta-analysis results showed that there was no difference between the two groups in the clinical pregnancy rate (OR = 0.97, 95% CI: 0.73-1.29, *P* = 0.82; **Figure 5A**).

#### Subgroup analysis

Five studies provided comparison data of clinical pregnancy rate in the fresh embryo transfer. There was low heterogeneity between the two groups (*P* = 0.65, I^2 = ^0%). The results of the meta-analysis showed that there was no difference between the two groups in the clinical pregnancy rate (OR = 0.97, 95% CI: 0.69-1.36, *P* = 0.86; **Figure 5B**).

Three studies provided data regarding frozen embryo transfer. There was moderate heterogeneity between the two groups (*P* = 0.09, I^2 = ^58%), and a random effects model analysis was used. The OR was 0.96 (95% CI, 0.38-2.44, *P* = 0.94; **Figure 5C**). These findings suggest that the type of embryo transfer did not significantly affect the clinical pregnancy rate in patients with prior SARS-CoV-2 infection.

### Secondary outcomes

#### Implantation rate

Four studies including 904 embryos transferred (158 embryos transferred from patients with COVID-19 infection and 746 embryos from controls) reported implantation rate. There was low heterogeneity between the two groups (*P* = 0.17, I^2 = ^41%), and a fixed effects model analysis was used. Meta-analyses of these studies showed no significant difference between the COVID-19 infection and control groups (OR 0.99, 95% CI 0.67-1.46, *P* = 0.96; **Figure 5D**) suggesting that COVID-19 infection does not affect the implantation rate in IVF treatment.

#### Early miscarriage rate

Three studies including 99 transfer cycles in the infection group and 223 transfer cycles in the control group have investigated the early miscarriage rate. There was low heterogeneity between the two groups (*P* = 0.19, I^2 = ^39%), and a fixed effects model analysis was used. No difference was found in the early miscarriage rate between the COVID-19 infection and control groups (OR 0.64, 95% CI 0.15-2.65, *P* = 0.53; **Figure 5E**), suggesting that COVID-19 infection does not affect the early miscarriage rate in IVF treatment.

## Discussion

The SARS-CoV-2 virus remains a significant global public health concern. In the early stage of the pandemic, the American Society for Reproductive Medicine (ASRM) and the European Society of Human Reproduction and Embryology (ESHRE), independently recommended suspending fertility services except for the most urgent cases (31, 32). More recently, with increased knowledge of SARS-CoV-2 and its transmission, reproductive care has gradually resumed within certain restrictions (33). However, there are insufficient data to show that SARS-CoV-2 infection negatively influences clinical outcomes in patients undergoing IVF treatments.

The results of this study indicate that past infection with SARS-CoV-2 had no impact on IVF treatment outcomes in terms of clinical pregnancy rate, implantation rate, and miscarriage rate. No significant difference was found in the subgroup analysis of clinical pregnancy rate for fresh and frozen embryo transfers.

Due to the outbreak of a new virus, little is known about the pathophysiology of SARS-CoV-2 and its potential impact on human endometrial and early embryo attachment. Significant progress has been made in understanding the molecular machinery of virus entry into host cells (1–3). However, contradictory data are available on the expression, interaction, and function of ACE together with another TMPRSS2 in human endometrial receptivity and early embryo implantation. A recent transcriptomic analysis indicated that the expression of ACE2 was significantly higher in the implantation window and TMPRSS2 increased during embryo implantation (12). On the contrary, another study suggested a low level of ACE1, ACE2, and TMPRSS2 in human endometrial cells at the transcripts level (13). Especially, the co-expression of ACE2 and TMPRSS2 proteins in human mature oocytes and preimplantation embryos (11, 34), and the expression of genes required for SARS-CoV-2 infection in trophectoderm cells (35), further increase the potential risk of SARS-CoV-2 infection on embryo survival and implantation. It has also been supposed that couples infected with SARS-CoV-2 may have poor reproductive outcomes after IVF treatment.

Several studies have examined the impact of SARS-CoV-2 on ovarian function during stimulation. In a small study including nine women with past infection undergoing oocyte retrieval, no difference in the levels of serum estradiol on the day of ovulation trigger, and serum progesterone on the day of oocyte retrieval, the ratios of serum estradiol/oocyte, and oocytes/follicles aspirated was reported when compared to the non-exposed group (36). However, another study reported a negative effect on oocyte yield in women who had a past SARS-CoV-2 infection more than 180 days before oocyte retrieval, compared to those had, who had a past infection 90-180 days and ≤90 days (25). The authors pointed out that their results need to be considered with caution as the sample size of the study was small (25).

As for the risk of vertical transmission of virus infection through gametes or IVF, a recently published study examined the viral RNA of SARS-CoV-2 in oocytes from women who were positive on the day of oocyte collection and found that the viral RNA was not detected in all oocytes (37). Up until now, several studies have reported that no viral RNA was found in follicular fluid (38–40), cumulus cells (39), ovarian medulla (40), vaginal secretions (40–42), and endometrial tissue (39) in SARS-CoV-2 positive women. Based on the above research results, the ovary, uterus, and genital tract are considered to be at low risk of SARS-CoV-2 infection.

To our knowledge, this systematic review is the first study to examine the effect of a history of SARS-CoV-2 infection on the clinical outcomes of fresh and frozen ET cycles. The study was conducted using a prospectively registered protocol and a comprehensive search strategy. The main strengths of the present study included a large number of patients, 317 patients with COVID-19 infection, and 904 controls undergoing IVF treatment, including 8 studies. The comparisons were performed not only for the main outcomes but also according to transfer type. Furthermore, in this review, we strictly followed the reporting guidelines while searching databases, selecting eligible articles, assessing quality, and analyzing the data.

There are still several limitations in the current study. First, the number of included studies was relatively small and the quality of included data was medium because most of the included studies are retrospective designs. Therefore, we conducted sensitivity analyses by excluding one study to evaluate the robustness of the effect size. The results indicated that excluding any single study had no significant effect on the total effect size. Second, the included patients exhibited heterogeneity in the baseline characteristics, such as time of COVID-19 diagnosis, detection methods, and severity of COVID-19, which could represent confounding factors and affect the outcomes. Our meta-analysis showed that a major of the results had low heterogeneity, and only the groups of frozen embryo transfers had moderate heterogeneity. To avoid the effect of moderate heterogeneity, a random effect model analysis was employed. A further limitation was that the time intervals between SARS-CoV-2 infection and IVF treatment exist differences in participants, which may not reflect the true effect of past SARS-CoV-2 infection on IVF outcomes. Finally, the potential limitation of this meta-analysis was the absence of data on live birth outcomes. Further detailed research is needed to investigate the long-term effects of SARS-CoV-2 infection on infertility treatment outcomes.

## Conclusion

In this systematic review and meta-analysis, past SARS-CoV-2 infection did not appear to harm the clinical outcomes of patients with a history of SARS-CoV-2 infection undergoing IVF treatment. The results can provide evidence for healthcare professionals who suggest treatment interventions and for couples who contemplate pregnancy through IVF. Further studies are warranted to further confirm these findings.

## Author contributions

YXu and KL conceived the concept and drafted the original manuscript. YXu, YXi, XC, and KL assessed the literature and discussed the manuscript. YXu and KL revised and edited the work. All authors contributed to the article and approved the submitted version.
